# Effectiveness of sodium bicarbonate and zinc chloride mouthwashes in the treatment of oral mucositis and quality of life in patients with cancer under chemotherapy

**DOI:** 10.1002/nop2.1168

**Published:** 2022-02-16

**Authors:** Fateme Mohammadi, Khodayar Oshvandi, Seyed Ramesh Kamallan, Salman Khazaei, Hossein Ranjbar, Fatemeh Ahmadi‐Motamayel, Mark Gillespie, Ensiyeh Jenabi, Seyed Yaser Vafaei

**Affiliations:** ^1^ 48430 Department of Pediatric Nursing, Chronic Diseases (Home Care) Research Center and Autism Spectrum Disorders Research Center Department of Nursing Hamadan University of Medical Sciences Hamadan Iran; ^2^ 48430 Department of Medical Surgical Nursing School of Nursing and Midwifery, Mother and Child Care Research Center Hamadan University of Medical Sciences Hamadan Iran; ^3^ 48430 Department of Medical‐Surgical Nursing Student Research Center Hamadan University of Medical Sciences Hamadan Iran; ^4^ Department of Epidemiology Health Sciences Research Center Health Sciences & Technology Research Institute Hamadan University of Medical Sciences Hamadan Iran; ^5^ 48430 Department of Hematology and Oncology Department of Internal Medicine School of Medicine Hamadan University of Medical Sciences Hamadan Iran; ^6^ 48430 Department of Oral and Maxillofacial Medicine School of Dentistry Dental Research Center Hamadan University of Medical Sciences Hamadan Iran; ^7^ 6413 School of Health Nursing and Midwifery University of the West of Scotland Paisley Scotland; ^8^ Research Assistant Professor of Reproductive Health (By Research) Autism Spectrum disorders Research Center Hamadan University of Medical Sciences Hamadan Iran; ^9^ 48430 Department of Pharmaceutics Department of Pharmaceutics and Pharmaceutical Biotechnology School of Pharmacy Hamadan University of Medical Sciences Hamadan Iran

**Keywords:** cancer, chemotherapy, oral mucositis, patients, sodium bicarbonate, zinc chloride

## Abstract

**Aim:**

The purpose of the study is to evaluate the effectiveness of sodium bicarbonate and zinc chloride mouthwashes on oral mucositis and quality of life in patients undergoing chemotherapy.

**Design:**

The present study was a randomized controlled trial study.

**Methods:**

One hundred forty‐four patients with a cancer diagnosis were randomly assigned into three groups: sodium bicarbonate mouthwash (*n* = 48), zinc chloride mouthwash (*n* = 48) and placebo group (*n* = 48). The severity of mucositis and quality of life were examined blindly at the baseline and 3‐week follow‐up.

**Results:**

The grade of oral mucositis decreased at the end of the third weeks in the sodium bicarbonate and zinc chloride groups rather than the placebo group (*p* < .001). The severity of oral mucositis in the sodium bicarbonate and zinc chloride groups decreased from end of the first week until third week (*p* < .001). In addition, there was significant difference in the severity of oral mucositis among the groups at the end of the second (*p* = .014) and the third weeks (*p* < .001). Also, there was a statistically significant difference in quality of life scores between the sodium bicarbonate and zinc chloride mouthwash with the placebo group (*p* < .001).

**Conclusion:**

Zinc chloride and sodium bicarbonate mouthwashes were effective in treating and reducing the severity of oral mucositis, and subsequently improving quality of life in patients with cancer under chemotherapy. Therefore, we can recommend zinc chloride and sodium bicarbonate at the beginning of chemotherapy to improve oral health and promoting quality of life in these patients.

## 1 INTRODUCTION

Cancer is one of the most common and debilitating diseases of this century, affecting 15 million people worldwide in 2020 (Zhang et al., 2017), with more than 7 million people dying of various types of cancer each year (Nipp et al., 2017). There are currently about 112,000 people with cancer in Iran, of which 54% are male and 46% are female (Kunitake et al., 2017); the majority of these patients have breast, colorectal, prostate, stomach or blood cancers (Kunitake et al., 2017). Responding to the growing prevalence of cancer in Iran has included the promotion of strategies guided by three key themes: prevention, early diagnosis and effective treatment with the least complications (Yavari et al., 2006).

The strategy encouraging effective treatment with the least complications emphasizes the importance of paying attention to the selection of appropriate treatment by the physician and the delivery of evidence‐based care by the nurse in order to reduce the likelihood of treatment complications (Rambod et al., 2018; Yavari et al., 2006). Chemotherapy is the most common treatment for various types of cancer (Rosenberg et al., 2013). But chemotherapy is associated with many problems and side effects (Pamungkas et al., 2017). Oral mucositis has been identified as one of the most debilitating and painful side effects of chemotherapy during treatment (Ertekin et al., 2004). This painful inflammation, accompanied by multiple ulcers of the oral mucositis, occurs in 40%–80% of patients undergoing chemotherapy (Ertekin et al., 2004; Mehdipour et al., 2011; Umeta et al., 2000) and causes difficulty in eating and drinking, speech impairment and persistent pain in the oral cavity. This can have significant consequences for the nutritional status and physical condition of these patients (Mehdipour et al., 2011; Rambod et al., 2018) and can consequently affect their mental health and quality of life (Mehdipour et al., 2011; Nipp et al., 2017; Rambod et al., 2018).

Despite the high incidence of oral mucositis and its acute and debilitating complications for these patients, there is no defined treatment available for mucositis, and significant efforts to develop supportive therapies have been made to reduce pain associated with the condition (Cirillo et al., 2015). In recent years, several studies have examined the prevalence of oral mucositis in patients undergoing chemotherapy and have tried to identify a way to treat oral mucositis for this group (Cabrera‐Jaimea, 2018; Mehdipour et al., 2011; Rambod et al., 2018). Studies that have examined the effectiveness of sodium bicarbonate on oral mucositis for this client group suggest that drugs with a 5% sodium bicarbonate base can be generally effective in the prevention and treatment of oral mucositis in cancer patients undergoing chemotherapy (Cabrera‐Jaimea, 2018). A study by Cobra et al. (2018) showed that both 5% and 10% sodium bicarbonate solution are effective in preventing oral mucositis in cancer patients undergoing chemotherapy. Mouthwash with 10% sodium bicarbonate solution was more effective in preventing oral mucositis than a 5% solution, but patients were less inclined to use the 10% solution citing an unpleasant odour and the generation of severe nausea (Cabrera‐Jaimea, 2018). The study by Cobra et al. highlights an important issue around administration of the mouthwash, as in most studies in this field, one or two vials of 5% sodium bicarbonate with non‐injectable normal saline have been prescribed to prevent oral mucositis to a group of cancer patients undergoing chemotherapy or radiotherapy. The patients themselves have been asked to combine and use these solutions every day (Cabrera‐Jaimea, 2018; Hamza et al., 2020; Najafi et al., 2016). Clearly, the use of solutions that are not generated on clinical and pharmaceutical principles in clean rooms and by pharmaceutical companies increases the risk of infection and oral mucositis in cancer patients, who are very vulnerable. Additionally, in order to reduce the potential for infection, unused 5% sodium bicarbonate and non‐injectable normal saline solutions should be discarded after 24 hr. This has cost implications for patients and the healthcare provider. These issues suggest that in order to create a palatable, financially viable and effective mouthwash requires consideration of the use of sodium bicarbonate 5% solution, developed by a pharmacist in a sterile environment.

Alternatively, several studies have examined the effect of zinc‐based drugs on oral mucositis (Ertekin et al., 2004; Mehdipour et al., 2011; Umeta et al., 2000) because zinc plays a vital role in promoting regeneration processes, cell membrane stability and membrane wound healing by increasing protein synthesis and nucleic acid as well as improving oxygen transfer (Ertekin et al., 2004). These studies have shown the effectiveness of zinc in the prevention and treatment of oral mucositis in cancer patients undergoing chemotherapy (Ertekin et al., 2004; Rambod et al., 2018), but Mansouri et al. (2012) argued that there was no benefit when oral zinc sulphate was prescribed for the treatment of high‐dose chemotherapy‐induced oral mucositis. Thus, efficacy and safety of oral zinc sulphate for chemotherapy‐induced oral mucositis remain controversial Mansouri et al., 2012). On the other hand, zinc is mostly used in the form of zinc sulphate capsules to treat oral mucositis, and due to severe nausea, patients undergoing chemotherapy are often reluctant to take zinc sulphate capsules and prefer to use mouthwashes over oral medications (Oshvandi et al., 2021; Tian, 2018).

The researchers therefore decided to study the effect of zinc chloride and sodium bicarbonate mouthwash on the treatment of oral mucositis and subsequently on quality of life in patients with cancer undergoing chemotherapy. The results of this research would identify the most effective treatment approach and shape future care delivery for this client group, influencing selection of the most appropriate mouthwash for the treatment of oral mucositis in these patients.

## 2 METHOD

### 2.1 Study design and participants

The present study was a randomized controlled trial with three parallel groups, two interventions and a control group conducted in a double‐blind manner in one oncology clinic affiliated to the University of Medical Sciences in western Iran from January 2019–August 2020. This study design was approved by the Ethics Committee of the Hamadan University of Medical Sciences (Umsha.rec.1399.651) and recorded at the Clinical Trials Center (IRCT 20190703044082N3).

The written informed consent was obtained from all the participants after providing them with sufficient information on the study. Inclusion criteria included: Patients who were in the stages 1–2 of cancer according to the medical record and the opinion of the treating physician and were deemed candidates for receiving the “3 + 7” chemotherapy regimen (3 days of the antibiotic daunorubicin and 7 days of the antimetabolite cytarabine), age above 18 years, receiving chemotherapy for the first time, mucositis incidence during the study, lack of pregnancy, lack of breastfeeding, lack of underlying diseases (diabetes as well as liver, renal and digestive diseases), lack of immune deficiency and lack of oral cavity disease prior to the start of chemotherapy and willingness to participate in the study. Exclusion criteria were thrombocytopenia (platelets less than 20,000 per mm^3^), history of smoking, worsening of the patient's condition and treatment discontinue for any reason.

### 2.2 Sample size

The sample size for this study calculated based on Chou et al study with power of 80% and *α* = 0.05 by using the formula (Choi & Kim, 2012). Therefore, about 44 patients was estimated for each group, which with the loss of about 10% of the samples during the study, sample size was finally considered 48 people in each group,

$$n=\frac{{\left(Z_{1‐\alpha /2}+Z_{1‐\beta }\right)}^{2}\left[p_{1}\;\left(1‐p_{1}\right)+p_{2}\left(1‐p_{2}\right)\right]}{{\left(p_{2}‐p_{1}\right)}^{2}}\times \sqrt{2}$$

alpha = 0.0500 (two sided); power = 0.8000; *p*_1_ = 0.2500; *p*_2_ = 0.6250; *n*2/*n*1 = 1.00. Estimated required sample sizes: *n*1 = 48; *n*2 = 48; *n*3 = 48.

### 2.3 Recruitment and allocation

After determining the sample size, a total of 154 patients were screened for eligibility; 10 patients were ineligible; and finally, 144 patients who gave written informed consent were enrolled and randomly assigned to one of the three groups (zinc chloride 0.2% mouthwash, 48 subjects; sodium bicarbonate 5% mouthwash, 48 subjects; and control, placebo mouthwash, 48 subjects) using block randomization with a volume of 3 and an allocation ratio of 1:1:1 using a computer‐generated randomization schedule, stratified by parity (two strata: first, and second or third). Sequentially numbered, opaque, sealed bottles with similar shape, colour and size containing of zinc chloride mouthwash, sodium bicarbonate mouthwash and placebo were used to conceal the allocation sequence and to maintain blinding, and necessary measures were taken, so that the sequence would remain concealed until the intervention was allocated to the intervention groups. The allocation sequence and packages were prepared by a person not involved in the recruitment, data collection and analysis.

### 2.4 Outcomes and data collection

In order to control the confounding variables, some actions were taken. A review of the literature suggests that low age and female gender are among the factors influencing the incidence rate of mucositis (Khanjani Pour‐Fard‐Pachekenari et al., 2019; Yarom et al., 2013). These variables were controlled by matching all the three groups and through statistical analyses. Also, oral health and salivary gland function are of the effective factors in the risk of developing mucositis (Khanjani Pour‐Fard‐Pachekenari et al., 2019). In order to control this confounding variable, the patients were examined by physicians before entering the study, and those with oral and dental problems were excluded. Furthermore, considering that the type of chemotherapy treatment given is known to promote the development of mucositis (Khanjani Pour‐Fard‐Pachekenari et al., 2019; Yarom et al., 2013), all the patients were under the same chemotherapy.

The primary outcome of the study was oral mucositis, evaluated at four time points: baseline and during the first, second and third weeks of intervention. The severity of oral mucositis was measured using the World Health Organization criteria for grading of oral mucositis. This scale measures mucositis at 5 levels (0–4). Level zero (lack of ulcers), stage one (pain and erythema), level two (erythema and ulcer), level three (ulcer and large erythema where the patient does not have the ability to eat solid food), level four (mucositis is so severe that cannot be easily repaired, and oral feeding is not possible). This scale has content and face validity and internal reliability of 0.91 in Iranian society (Ghadiri et al., 2014; Khanjani Pour‐Fard‐Pachekenari et al., 2019).

The secondary outcome was quality of life the patients who evaluated at two time points (baseline and 3 weeks after the intervention) by using the European Organization for Research and Treatment of Cancer Quality of Life Questionnaire (EORTC QLQ‐C30). This questionnaire has 30 items in five functional dimensions (physical, role, cognitive, emotional and social), nine symptom dimensions (fatigue, pain, nausea‐vomiting, dyspnoea, appetite loss, sleep disturbance, constipation‐diarrhoea and financial problems) and one general dimension health and quality of life. Accordingly, all domains receive scores between 0–100. In the five functional dimensions and the general dimension health and quality of life, a higher score indicates a better condition of the person, but in the nine symptom dimensions, a higher score indicates more symptoms and problems of the disease in the individual. This questionnaire has appropriate validity and reliability in patients with cancer in Iran (Montazeri et al., 2000). Also, some demographic information of the patients was collected using a demographic information checklist. In order to avoid bias in collecting and completing the questionnaires, the assistant researcher was used, who was not aware of the allocation of individuals in the intervention and control groups. Assistant researcher was a clinical nurse in oncology clinic with ten years of experience, who observed patients' mouths and completed the World Health Organization criteria in the baseline, first, second and third weeks for each patient and recorded mucositis grade. Also, the quality of life questionnaire was completed by patients at the beginning and end of the study (third week) then was collected by the assistant researcher.

### 2.5 Interventions

Mouthwashes for all three groups of zinc chloride, sodium bicarbonate and placebo in this study were prepared by a pharmacist specializing in pharmaceutics in the clean room of the University of Medical Sciences in Western Iran. Mouthwash for the sodium bicarbonate group contains sodium bicarbonate 5%, greasy mint, preservative; mouthwash for the zinc chloride group contains zinc chloride 0.2%, greasy mint, preservative; and mouthwash for the control group contains sterile water, greasy mint and preservative. All patients in three groups were educated in routine oral care (teeth brushing, proper nutrition and mouth hygiene). The patients in each group should rinse their mouths every 8 hr 2 times and each time 2 min with 7.5 ml from mouthwash. The time interval between each mouthwash was 15 min. Participants in the 3 groups rinsed their mouth, so that the mouthwash covered the tongue, palate, throat, inside the cheeks and all the tissues of the mouth well.

### 2.6 Data analysis

Data analyses were conducted using SPSS version 22. P values of less than 0.05 were considered statistically significant. To analyze the data, descriptive statistics (namely frequency, percentage, mean and standard deviation) were used and inferential statistics including the chi‐squared and Fisher's exact test (comparison of data distribution), covariance (adjustment of confounding variables), Kruskal–Wallis test (comparison oral mucositis among groups in non‐normal distribution) and Friedman's nonparametric test (comparison of oral mucositis intragroup). The one‐way was used for analysis of variance (comparison of quality of life among groups) and the dependent *t* test comparison of quality of life (intragroup comparison).

## 3 RESULTS

The average age of patients participating in this study was 46.73 ± 2.41 years in the sodium bicarbonate, 47.04 ± 2.53 years in the zinc chloride group and 46.12 ± 2.43 years in the placebo. Most of the patients in three groups were male, married, and 28 (58.33%) had diploma education. Most had an average income of 500 Dollars. Moreover, the majority of patients in all three groups suffered from grade 1 oral mucositis. The results showed that there was no statistically significant difference among the three groups in terms of demographic information (Table 1).

**TABLE 1:** Demographic information of this study participants

Demographic variables		Sodium bicarbonate group *N* (%)	Zinc chloride group *N* (%)	Placebo group *N* (%)	χ2 *p* Value
Age	23–33	8 (16/67)	9 (18/75)	9 (18/75)	χ^2^ = 2.87, *p* = .813^a^
	34–44	15 (31/25)	15 (31/25)	14 (29/17)	
	45–55	18 (37/5)	19 (39/58)	19 (39/58)	
	56–66	7 (14/58)	5 (10/41)	6 (12/5)	
Gender	Female	21 (34/75)	22 (45/84)	22 (45/84)	χ^2^ = 3.42, *p *= .912^a^
	Male	27 (56/24)	26 (54/16)	26 (54/16)	
Marital status	Single	12 (25)	13 (27/09)	11 (22/91)	χ^2^ = 3.76, *p *= .811^a^
	Married	36 (75)	35 (72/91)	37 (77/09)	
Education	Illiterate	4 (8/34)	5 (10/41)	5 (10/41)	χ^2^ = 3.05, *p *= .843^b^
	Primary	8 (16/67)	7 (14/58)	6 (12/5)	
	Diploma	28 (58/33)	28 (58/33)	28 (58/33)	
	Bachelor	7 (14/58)	5 (10/41)	6 (12/5)	
	Master degree and higher	1 (2/087)	3 (6/25)	3 (6/25)	
Cancer type	Liver	8 (16/67)	9 (18/75)	8 (16/67)	χ^2^ = 1.02, *p *= .822^b^
	Stomach	6 (12/5)	6 (12/5)	8 (16/67)	
	Colon	6 (12/5)	3 (6/25)	4 (8/34)	
	Uterus	4 (8/34)	4 (8/34)	5 (10/41)	
	Breast	3 (6/25)	3 (6/25)	4 (8/34)	
	Kidney	6 (12/5)	6 (12/5)	4 (8/34)	
	Bladder	5 (10/41)	5 (10/41)	4 (8/34)	
	Lung	5 (10/41)	6 (12/5)	6 (12/5)	
	Leukaemia	5 (10/41)	5 (10/41)	5 (10/41)	
Job	Self‐employed	14 (29/17)	13 (27/09)	15 (31/25)	χ^2^ = 1.87, *p *= .604^a^
	Employee	13 (27/09)	14 (29/17)	13 (27/09)	
	livestock and farmer	14 (29/17)	14 (29/17)	15 (31/25)	
	Housewife	7 (14/58)	7 (14/58)	5 (10/41)	
Blood factors^c^	WBC	10.64 ± 3.18	10.74 ± 3.0.13	10.781 ± 3.22	*F* = 3.54, *p *= .784^a^
	RBC	4.78 ± 2.31	4.81 ± 2.62	4.98 ± 2.41	
	PLT	198 ± 11.31	196 ± 12.78	198 ± 12.21	
	HB	14.04 ± 2.27	13.74 ± 2.24	14.12 ± 2.78	

Note

Values are expressed as no. (%).

^a^ Chi‐square test.

^b^ Fisher's exact test.

^c^ ANOVA test.

**FIGURE 1:**
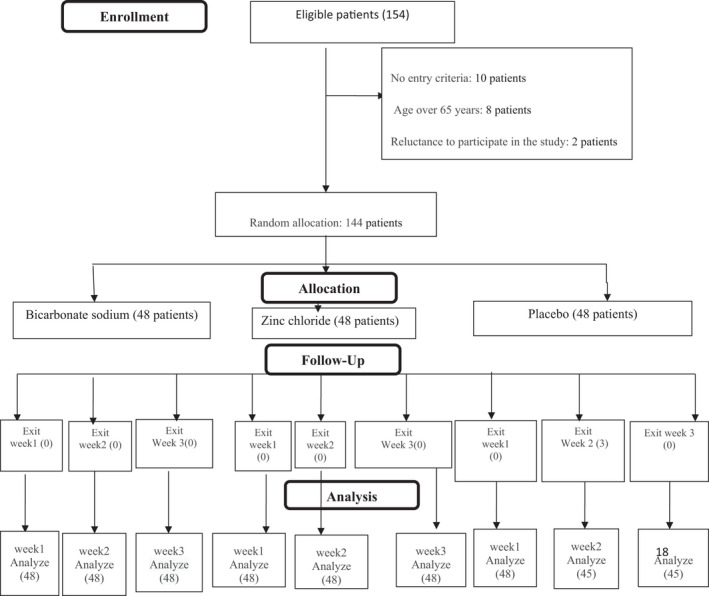
Flow diagram of the study

One hundred fifty‐four patients with cancer were present in the oncology clinic, 10 of whom had not the inclusion criteria, so 144 patients arrived to the study (48 patients in each group). Three patients in the placebo group left the study due to unwillingness to continue during the first week. So, 48 patients in the sodium bicarbonate group, 48 patients in the zinc chloride group and 45 patients in the placebo group completed this study (Figure 1).

An investigation of different grades of oral mucositis in the study between groups showed that none of the patients participating in the study were affected by the grade 4 mucositis. The prevalence of grades of oral mucositis between groups was significant at the end of the second (*p* <.002) and third (*p* <.001) weeks. Accordingly, the oral mucositis with 1 and 2 grades in the bicarbonate and zinc chloride groups had started to decrease in the first week then continued until the third week. In the third week, all patients with grade 1 and 2 oral mucositis improved in these two groups, and small number of patients with grade 3 mucositis in these two groups did not recover in the end of the third week. Also, the zinc chloride group had better performance in treatment and reduction of oral mucositis intensity than the sodium bicarbonate group (Table 2).

**TABLE 2:** Oral mucositis grades between groups during this study

Mucositis grade		Sodium bicarbonate group *N* (%)	Zinc chloride group *N* (%)	Placebo group *N* (%)	*p* value
Study weeks					
Baseline	Grade 0	48 (100)	48 (100)	48 (100)	.894^a^
Week 1	Grade 0	42 (87.50)	43 (89.58)	38 (79.161)	.0823^a^
	Grade 1	6 (12.50)	5 (10.42)	10 (20.83)	
	Grade 2	0 (0)	0 (0)	0 (0)	
Week 2	Grade 0	38 (79.16)	40 (83.34)	27 (56.25)	.002^b^
	Grade 1	6 (12.50)	4 (8.33)	8 (16.67)	
	Grade 2	4 (8.33)	4 (8.33)	10 (20.83)	
	Grade 3	0 (0)	0 (0)	0 (0)	
Week 3	Grade 0	35 (72.92)	37 (77.08)	20 (41.67)	.001^b^
	Grade 1	7 (14.58)	6 (12.50)	10 (20.83)	
	Grade 2	3 (6.25)	4 (8.33)	10 (20.83)	
	Grade 3	3 (6.25)	1 (2.08)	5 (10.42)	

Note

Values are expressed as no. (%).

^a^ Chi‐square test.

^b^ Fisher's exact test.

Also, the results of the Freidman's test showed that the effect of time on oral mucositis severity of oral mucositis was significant in the sodium bicarbonate and zinc chloride groups (*p* <.001); accordingly, the severity of mucositis in the sodium bicarbonate and zinc chloride groups decreased from end of the first week until the third week. To compare the study groups at different time intervals, the results of the Kruskal–Wallis test showed that there was significant difference among the groups at the end of the second (*p* =.014) and the third weeks (*p* <.001; Table 3).

**TABLE 3:** Comparison of oral mucositis at different time points among groups

Groups	Baseline Mean (*SD*)	1 week after intervention Mean (*SD*)	2 weeks after intervention Mean (*SD*)	3 weeks after intervention Mean (*SD*)	*p* value
Sodium bicarbonate group	6.87 (1.12)	5.86 (1.44)	2.97 (1.04)	2.17 (1.12)	.001^a^
Zinc chloride group	6.98 (0.89)	5.65 (1.32)	3.15 (1.54)	2.55 (1.33)	.001^a^
Placebo group	6.89 (1.87)	6.69 (1.29)	7.38 (1.62)	5.38 (1.14)	.62^a^
*p* value	.98^b^	.58^b^	.014^b^	.001^b^	

^a^ The Friedman's test was used for comparison within groups.

^b^ The Kruskal–Wallis test was used for comparison between groups.

The quality of life in these patients was compared with one‐way ANOVA test. This test showed no significant difference among the three groups at the beginning of the study (*p* >.05), but at the end of the third week, a statistically significant difference was observed among the three groups (*p* <.001). Also, the results of the dependent *t* test showed that the effect of time on quality of life was significant for the sodium bicarbonate and zinc chloride groups (*p* <.001). Also, the zinc chloride group had better performance promoting the quality of life than the sodium bicarbonate group (Table 4).

**TABLE 4:** Comparison of quality of life at different time points among groups

Groups	Baseline mean (*SD*)	3 weeks after intervention Mean (*SD*)	*p* value
Sodium bicarbonate group	41.27 (1.65)	68.03 (1.32)	.001^b^
Zinc chloride group	40.89 (1.17)	70.61 (1.21)	.001^b^
Placebo group	41.24 (1.32)	40.98 (1.14)	.721^b^
*p* value	.97^a^	.001^a^	

^a^ One‐way ANOVA.

^b^ Dependent *t* test.

## 4 DISCUSSION

The results of the present study have revealed that both zinc chloride and sodium bicarbonate mouthwashes are effective in treating oral mucositis during the third week. Additionally, the findings of this study showed that there was a statistically significant difference among the two interventions with the placebo group in treatment and reduce the severity of oral mucositis. And, only one patient in the zinc chloride group and three patients in the sodium bicarbonate group showed grade 3 oral mucositis during the third week of follow‐up. Based on this, it seems that zinc chloride and sodium bicarbonate mouthwashes are both effective in treating oral mucositis and reducing the severity of oral mucosal in cancer patients undergoing.

Consistent with the findings of the this study, Chitapanarux et al. (2018) stated although the highest incidence, severity and pain of oral mucositis were reported in the second week in patients with cancer under chemotherapy combined with radiotherapy, sodium bicarbonate mouthwash 5% reduces the incidence, severity and pain of oral mucositis in the third and fourth weeks (Chitapanarux et al., 2018). However, in this study, only three patients showed oral mucositis with grade 3 in the sodium bicarbonate group. Better results can be due to the meticulous preparation of sodium bicarbonate mouthwash by the pharmacist in a cleanroom because in the study by Chitapanarux et al., a nurse mixed two tablespoons of sodium bicarbonate powder with one litre of water and provided it to patients. It is clear that principled and accurate preparation of mouthwash can be effective in the prevention and treatment of oral mucositis in these patients (Chitapanarux et al., 2018). David and Shree (2019) also stated that although sodium bicarbonate mouthwash has been able to reduce the severity of oral mucositis in patients undergoing chemotherapy so that most patients have mild to moderate mucositis with slight pain, over 7 days, there was no statistically significant difference in the incidence and treatment of oral mucositis in these patients. The difference from that study may be due to differences in the type of treatment of cancer patients, different assessment tools and the different duration of the study. David and Shree's (2019) result indicates the effectiveness of 5% sodium bicarbonate mouthwash in the prevention and treatment of oral mucositis in patients with acute head and neck malignancy. This finding is consistent with the present study (David & Shree, 2019). Cabrera‐Jaimea (2018) stated that sodium bicarbonate mouthwash could reduce the incidence, severity and level of oral mucosal pain in cancer patients undergoing chemotherapy. Therefore, sodium bicarbonate mouthwash has 10% better therapeutic and preventive effects (Cabrera‐Jaimea, 2018). However, the probable cause of increased severity of oral mucositis in their study compared with the present study may also be the severity of the disease (acute cervical tumour) and the groups under study. Also, in line with the findings of this study, other studies by Rambod et al. (2018), Mehdipour et al. (2011) and Ertekin et al. (2004) state that the drug compounds in the form of capsules and mouthwash with a zinc base are remarkably effective in the prevention and treatment of oral mucositis in cancer patients receiving treatment (Ertekin et al., 2004; Mehdipour et al., 2011; Rambod et al., 2018) because zinc plays a vital role in improving physiological processes in the body like growth and development, immune system durability, cell membrane stability and wound healing by increasing protein and nucleic acid synthesis as well as improving oxygen transport. Therefore, it can preserve cell membranes in cancer patients undergoing chemotherapy or radiotherapy and improve the function of cells, especially mucous membrane cells, and reduce their destruction; thus, they are very effective in treating oral mucositis (Mehdipour et al., 2011). In this regard, Rambod et al. state that zinc sulphate capsules have highly reduced the severity of oral mucositis in patients with leukaemia undergoing chemotherapy (Rambod et al., 2018). However, zinc chloride mouthwash was effective in the treatment of oral mucositis in this study, and there was only one patient with grade 3 oral mucositis in the zinc chloride group across the duration of the study. A possible reason for this difference could be to the ease of the use of mouthwash instead of oral capsules. Because the majority of patients undergoing chemotherapy suffer from severe nausea, difficulty in swallowing and oral mucositis, swallowing a capsule is painful for them and, it is probably more comfortable for them to use mouthwash (Ertekin et al., 2004; Mehdipour et al., 2011; Mosalaei et al., 2010; Moslemi et al., 2016; Rambod et al., 2018). Also, Mehdipour et al. indicate that the severity of oral‐pharyngeal mucositis in patients undergoing radiotherapy in the zinc sulphate group is less than placebo (Mehdipour et al., 2011).

Other studies signify that there is a statistically significant difference in the mean of oral mucositis between the placebo and zinc sulphate groups (Lin et al., 2006; Moslemi et al., 2016). Therefore, these studies showed that zinc‐based drugs can be effective in the prevention and treatment of oral mucositis in cancer patients through promoting the activity of immune cells, preventing tissue damage, including oral mucositis, increasing protein synthesis and nucleic acid processes, as well as promoting tissue damage healing (Lin et al., 2006). Therefore, the use of drugs containing zinc, especially mouthwashes, can comprehensively reduce the incidence and severity of oral mucositis in cancer patients undergoing chemotherapy because it is easier to use (Ana et al., 2020; Hewlings & Kalman, 2020; de Menêses et al., 2020).

The most important finding of this study was the effectiveness of zinc chloride mouthwash compared with sodium bicarbonate in the treatment of oral mucositis. However, there is no study available to researchers that compare sodium bicarbonate and zinc chloride mouthwashes and investigates their effects on the treatment of oral mucositis. Therefore, comparing the effectiveness of sodium bicarbonate and zinc chloride with other drugs was investigated. In this regard, David and Shree (2019) also stated that although sodium bicarbonate mouthwash is rather effective in reducing the incidence and severity of oral mucositis in cancer patients undergoing radiotherapy, turmeric mouthwash proved to be more effective in treating and reducing the severity and level of oral mucosal pain (David & Shree, 2019). Chitapanarux et al., also stated in their study in 2018 that although sodium bicarbonate mouthwash is effective in the treatment of oral mucositis, benzydamine hydrochloride has been more effective in treating and reducing the severity and pain of oral mucositis (*p* <.05) (Chitapanarux et al., 2018). This is consistent with the findings of the present study. However, there was no difference between the two groups in controlling the pain caused by oral mucositis because, in the present study, sodium bicarbonate mouthwash was effective in treating and reducing the severity of oral mucositis, though less effective than zinc chloride. However, unlike the present study, there was no difference between the two groups in terms of controlling oral mucosal pain in the above study (*p* =.80). This difference can be rooted in diversity in the type of medications used as well as the community under investigation. In line with the findings of the present study, Alkhouli et al. (2019) in their study state that sodium bicarbonate mouthwash is successful in treating and reducing oral mucosal pain in children with leukaemia, but olive oil has more therapeutic effects than sodium bicarbonate (*p* =.0) (Alkhouli et al., 2019).

In this study also, quality of life patients under chemotherapy improved in the sodium bicarbonate and zinc chloride groups than the placebo group during this study, even the zinc chloride group had better performance promoting the quality of life than the sodium bicarbonate group. Although, many studies have examined the quality of life in patients with cancer, but few studies have examined the effect of herbal and chemical drugs on the treatment of oral mucositis and consequently its effect on the quality of life in these patients. Obviously, when the severity of oral mucositis in these patients decreases, they report less pain, a greater desire and ability to eat, and better able to participate in self‐care. As a result, they experience a better quality of life (Bachok et al., 2018). In this regard, the study of Bachok et al. Oral7^®^ mouthwash (an immunologically active saliva substitute) showed that has reduced the severity of mucositis among head and neck cancer patients undergoing radiotherapy and improved their quality of life (Bachok et al., 2018). Also, Zhang et al. stated that Chinese herbal medicine mouthwash affected on reduced mucosal severity and improved quality of life among cancer patients undergoing chemotherapy (Zhang et al., 2020). Cabrera et al. presented sodium bicarbonate reduced oral mucositis and improved quality of life among cancer patients undergoing chemotherapy (Cabrera‐Jaimea, 2018). Khanjani Pour et al. also stated that honey mouthwash is effective in treating oral mucositis and improving the quality of life of patients with leukaemia undergoing chemotherapy (Khanjani Pour, 2018).

### 4.1 Limitations of the Study

One of the notable limitations of the present study was the small sample size of the participants in the study. Therefore, it is recommended to conduct similar studies in the coming years in different communities and with larger sample size. Through that, a more precise estimation of the effectiveness of zinc chloride and sodium bicarbonate mouthwash 10% becomes possible in the treatment and reduction of the severity of oral mucositis and consequently oral health in cancer patients treated with chemotherapy. Moreover, in this study, only cancer patients undergoing chemotherapy were studied. It is suggested to investigate the effect of zinc chloride and sodium bicarbonate mouthwash on oral mucositis treatment as well as the easier use of mouthwash than capsule in cancer patients undergoing radiotherapy due to severe nausea in these patients and their swallowing problems. Another limitation was the evaluation of only oral mucositis in this study, so evaluating the effectiveness of mouthwashes on the treatment of mucositis in other mucous membranes of the gastrointestinal tract can provide more valuable information.

### 4.2 Implications for practice

Based on the study, zinc chloride and sodium bicarbonate mouthwash affected on treatment mucositis in the patients with cancer under chemotherapy. Moreover, the implication of the study for clinical practice is that zinc chloride and sodium bicarbonate mouthwash were safe and cost‐effective in these patients and could be easily used at the beginning of chemotherapy. It recommend zinc chloride and bicarbonate sodium as therapeutic care methods in clinical settings.

## 5 CONCLUSION

The results of this study showed that both zinc chloride and sodium bicarbonate mouthwashes are effective in treating and reducing the severity of oral mucositis in cancer patients undergoing chemotherapy and consequently improves the quality of life of these patients. So, we can recommend zinc chloride and bicarbonate sodium as therapeutic care methods in clinical settings at the beginning of chemotherapy for these patients to improve oral health and subsequently reduce the pain and suffering caused by the side effects of chemotherapy.

## CONFLICT OF INTEREST

The authors declare no competing interests.

## AUTHOR CONTRIBUTIONS

FM, SYV, KHO, SRK, HR and SKH were involved in the conception of the study and designed the study. They are responsible for data collection, data analysis and interpretation. FM, SYV, KHO, SRK, HR and SKH drafted the primary manuscript, revised and approved the final manuscript.

## ETHICAL APPROVAL

The study design was approved by the Ethics Committee of the Hamadan University of Medical Sciences (Umsha.rec.1399.651), and recorded at the Clinical Trials Center (IRCT 20190703044082N3). Also, at the beginning of study, the researcher introduced herself and explained the aims of this study, and informed consent was obtained after providing written explanations. The participants were assured that all information would remain confidential. The researcher created the opportunity for participants to inform the researcher about their withdrawal from the study at any stage of the study and assured them that their lack of participation or withdrawal would not have any consequences for them.

## CONSENT TO PARTICIPATE

The researchers provided the opportunity for participants to inform the researcher about their withdrawal from the study at any stage and assured them that their lack of participation or withdrawal would not have any consequence for them. Finally, participants gave written consent for their personal or clinical details along with any identifying images to be published in this study.

## CONSENT FOR PUBLICATION

Not applicable.

## Data Availability

The data sets used and/or analyzed during the current study is available from the corresponding author on reasonable request.
